# Caveolin 1 and 2 enhance the proliferative capacity of BCAM-positive corneal progenitors

**DOI:** 10.1038/s41598-024-81283-4

**Published:** 2025-02-24

**Authors:** Yuzuru Sasamoto, Shoko Kiritoshi, Catherine A. A. Lee, Yoshiko Fukuda, Gabrielle Martin, Bruce R. Ksander, Markus H. Frank, Natasha Y. Frank

**Affiliations:** 1 Division of Genetics, Brigham and Women’s Hospital, Boston, MA USA; 2 Transplant Research Program, Boston Children’s Hospital, Boston, MA USA; 3 Department of Ophthalmology, Chobanian & Avedisian School of Medicine, Boston University, Boston, MA USA; 4 Massachusetts Eye & Ear Infirmary, Schepens Eye Research Institute, Boston, MA USA; 5 Harvard Stem Cell Institute, Harvard University, Cambridge, MA USA; 6 Department of Dermatology, Harvard Skin Disease Research Center, Brigham and Women’s Hospital, Boston, MA USA; 7 School of Medical and Health Sciences, Edith Cowan University, Perth, WA Australia; 8 Department of Medicine, VA Boston Healthcare System, Boston, MA USA

**Keywords:** Caveolae, Caveolin, CAV1, CAV2, BCAM, Laminin α5, Laminin α3, FGFR2, Corneal epithelial progenitors ABCB5, Stem-cell research, Extracellular signalling molecules

## Abstract

Caveolin (CAV) 1 and 2 are integral membrane proteins that constitute major components of small membrane pouches termed caveolae. While several functions have been described in other tissues, the roles of CAV1 and CAV2 in the ocular surface have remained unknown. In the current study, we investigated the expression and function of CAV1 and CAV2 in the human cornea. We found CAV1 and CAV2 to be preferentially expressed by proliferative Basal Cell Adhesion Molecule (BCAM)-positive progenitor cells along the entire limbal and corneal basal epithelial layer. Functional gene knockdown studies reveal that BCAM, BCAM co-expressed Laminin α5 (LAMA5) and Laminin α3 (LAMA3) regulate expression of CAV2. Mechanistically, we demonstrate that CAV1 and CAV2 contribute to enhanced BCAM-positive cell proliferation through regulation of Fibroblast Growth Factor Receptor 2 (FGFR2) cell surface expression. In aggregate, our study identifies specific expression of CAV1 and CAV2 in BCAM-positive corneal basal epithelial cells and uncovers a novel CAV1/CAV2-dependent mechanism of corneal progenitor cell proliferation, with potential implications for therapeutic enhancement of corneal regeneration.

**Supplementary Information:**

The online version contains supplementary material available at 10.1038/s41598-024-81283-4.

## Introduction

The cornea is a critical component in the ocular system, serving as the primary barrier against environmental damage and contributing to the majority of the eye’s focusing power. Its maintenance and repair rely on a specific group of cells known as corneal progenitors that include limbal stem cells (LSCs) and transit amplifying cells (TACs) located in the basal epithetial layer of limbus and central cornea^1^. These cells play a crucial role in corneal homeostasis, wound healing, and regeneration, thereby contributing to the maintenance of clear vision.

ATP-binding cassette (ABC) superfamily member ABCB5 has been identified as the molecular marker for prospective isolation of LSCs capable of long-term corneal regeneration^2–9^. Our recent single-cell (sc) RNA-seq analyses of ABCB5-positive LSCs identified a subpopulation of cells with high proliferative capacity expressing the basal cell adhesion molecule (BCAM)^10,11^. Immunohistochemical studies revealed that in addition to LSCs, BCAM was expressed by the TACs located in the basal epithelial layer of the limbus and central cornea^10^. scRNA-seq analyses also revealed that the BCAM-positive cell cluster within ABCB5-positive LSCs exhibited higher levels of *CAV1* and *CAV2* mRNAs compared to ABCB5-positive BCAM-negative cells^10^. Observation of the high CAV1 and CAV2 expression levels in the BCAM-positive LSC subpopulation led us to hypothesize that these molecules might contribute to the enhanced proliferative capacity of BCAM-positive cells not only among the LSCs but also the TACs.

Caveolins are a family of integral membrane proteins, which form the principal components of caveolae^12,13^, flask-shaped vesicular invaginations of the plasma membrane with multiple cellular roles, including vesicular transport, signal transduction regulation and cholesterol transport^12,14–16^. Among the Caveolin family, CAV1, which is expressed in diverse tissues, has been shown to interact with various signal proteins, including G-proteins, epidermal growth factor receptor (EGFR), Src-like kinases, H-Ras, and endothelial nitric oxide synthase^17–22^. CAV2 is closely associated with CAV1, forming stable hetero-oligomeric complexes within the caveolae, and requires CAV1 for accurate localization within the plasma membrane^23–25^. The co-expression of CAV1 and CAV2 results in the formation of deeper caveolae compared to the expression of CAV1 alone^26^. Within the cornea, CAV1 expression has been detected in both the endothelium and the basal epithelial layer^17^. Further studies have indicated a correlation between aging and increased CAV1 expression levels^27^. However, the precise roles of CAV1 and CAV2 in the corneal epithelium remain to be elucidated.

In the current study, we demonstrate that CAV1 and CAV2 expressed in BCAM-positive progenitors along the corneal basal epithelial layer are essential for retaining the critical regulator of corneal differentiation Fibroblast Growth Factor Receptor 2 (FGFR2) on the cellular surface and for maintaining their proliferative capacity pointing to a novel role of CAV1 and CAV2 in human cornea.

## Results

### CAV1 and CAV2 are Expressed in BCAM-positive Basal Epithelial Cells in the Human Limbus and Cornea

**Fig. 1:**
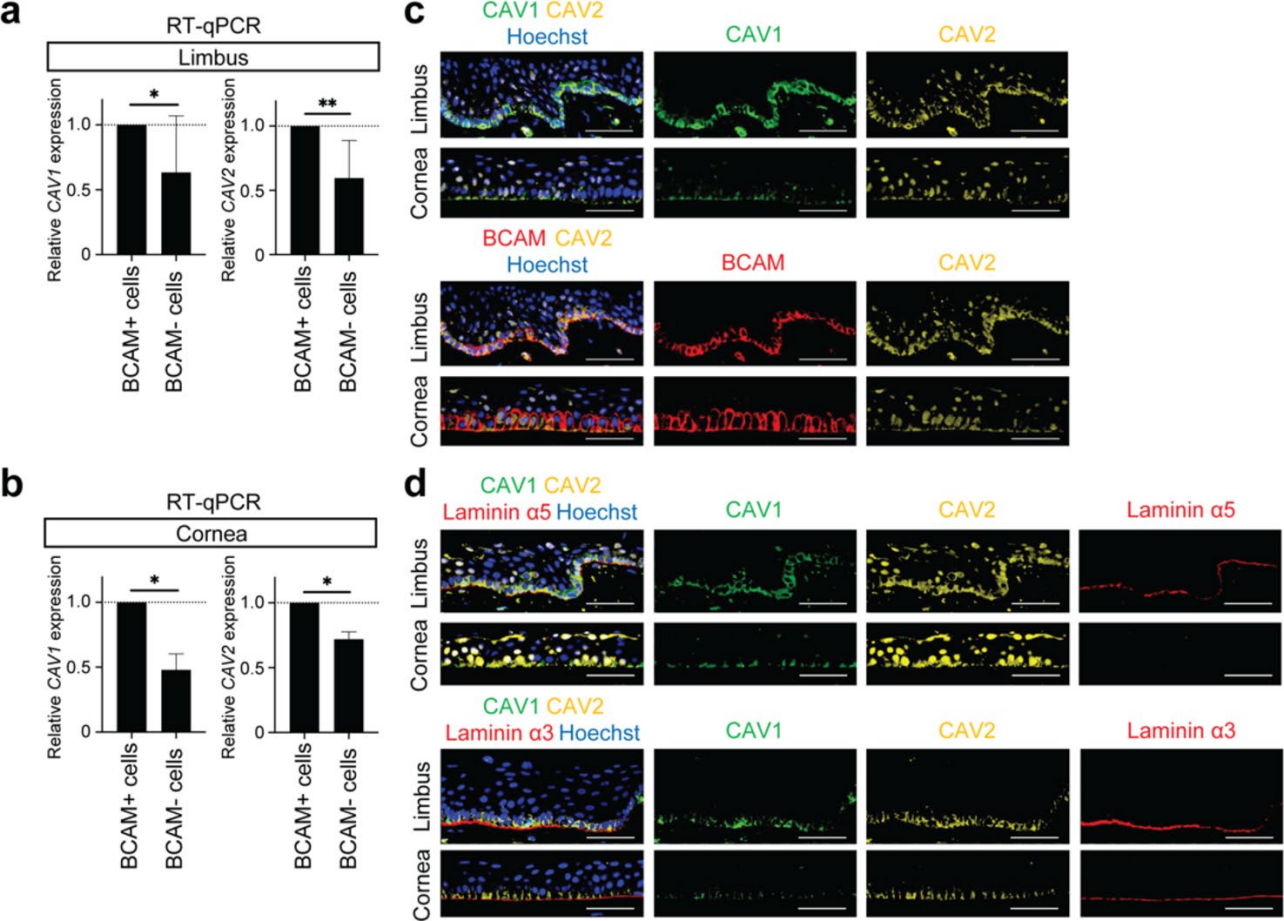
Evaluation of CAV1 and CAV2 expression in human limbus and cornea. Comparative analyses of *CAV1* and *CAV2* mRNA expression in BCAM-positive and BCAM-negative cells in the limbus (**a**) **(***n* = 8) and cornea (**b**) (*n* = 3) (*p < 0.05, **p < 0.01) (**c**) Representative immunostaining analyses of CAV1 (green), CAV2 (yellow) and BCAM (red) co-expression in the limbus and cornea. Sequential sections were used to illustrate BCAM and CAV1 co-expression since both antibodies were raised in rabbits. Nuclei are visualized with Hoechst 33342 (blue). *n* = 3. Scale bar, 50 μm. (**d**) Representative immunostaining analyses of CAV1 (green), CAV2 (yellow) and Laminin α5 (red) co-expression (top panels) and CAV1 (green), CAV2 (yellow) and Laminin α3 (red) (bottom panels) in the limbus and the cornea. Nuclei are visualized with Hoechst 33342 (blue). *n* = 3. Scale bar, 50 μm.

Based on our previous scRNA-seq studies demonstrating high levels of *CAV1* and *CAV2* in BCAM-positive cells within ABCB5-positive LSCs^10^, we hypothesized that *CAV1* and *CAV2* might also be enriched in BCAM-positive basal epithelial cells outside the LSC niche. RT-PCR analyses of sorted BCAM-positive and BCAM-negative cells isolated from the limbus and cornea revealed significantly lower *CAV1* and *CAV2* mRNA levels in BCAM-negative cells (Fig. 1a). In the limbus, *CAV1* expression was 36.6 ± 43.5% lower (*p* = 0.0492, *n* = 8) and *CAV2* expression 40.5 ± 29.3% lower (*p* = 0.0058, *n* = 8) in BCAM-negative cells. In the cornea, BCAM-negative cells expressed 52.2 ± 12.3% lower levels of *CAV1* and 28.1 ± 5.7% lower levels of *CAV2* (Fig. 1b). Immunofluorescent analyses of sequential corneal sections confirmed CAV1 and CAV2 co-expression with BCAM on the cell membrane of the basal epithelial cells from the limbus to the cornea (Fig. 1c). Of note, in some samples, nuclear, likely non-specific, staining of CAV2 was observed in both basal and suprabasal cells (Fig. 1c**)**. As previously reported, Laminin α5, a ligand of BCAM^28,29^, was detected by immunostaining specifically in the limbal basement membrane in close proximity to BCAM-expressing basal epithelial cells (Fig. 1d)^10^. Another major basement membrane component, Laminin α3 ^30,31^, was detected in the proximity of the BCAM-expressing cells along the entire basal epithelial layer (Fig. 1d).

### Regulation of CAV1 and CAV2 expression in Limbal epithelial cells

**Fig. 2:**
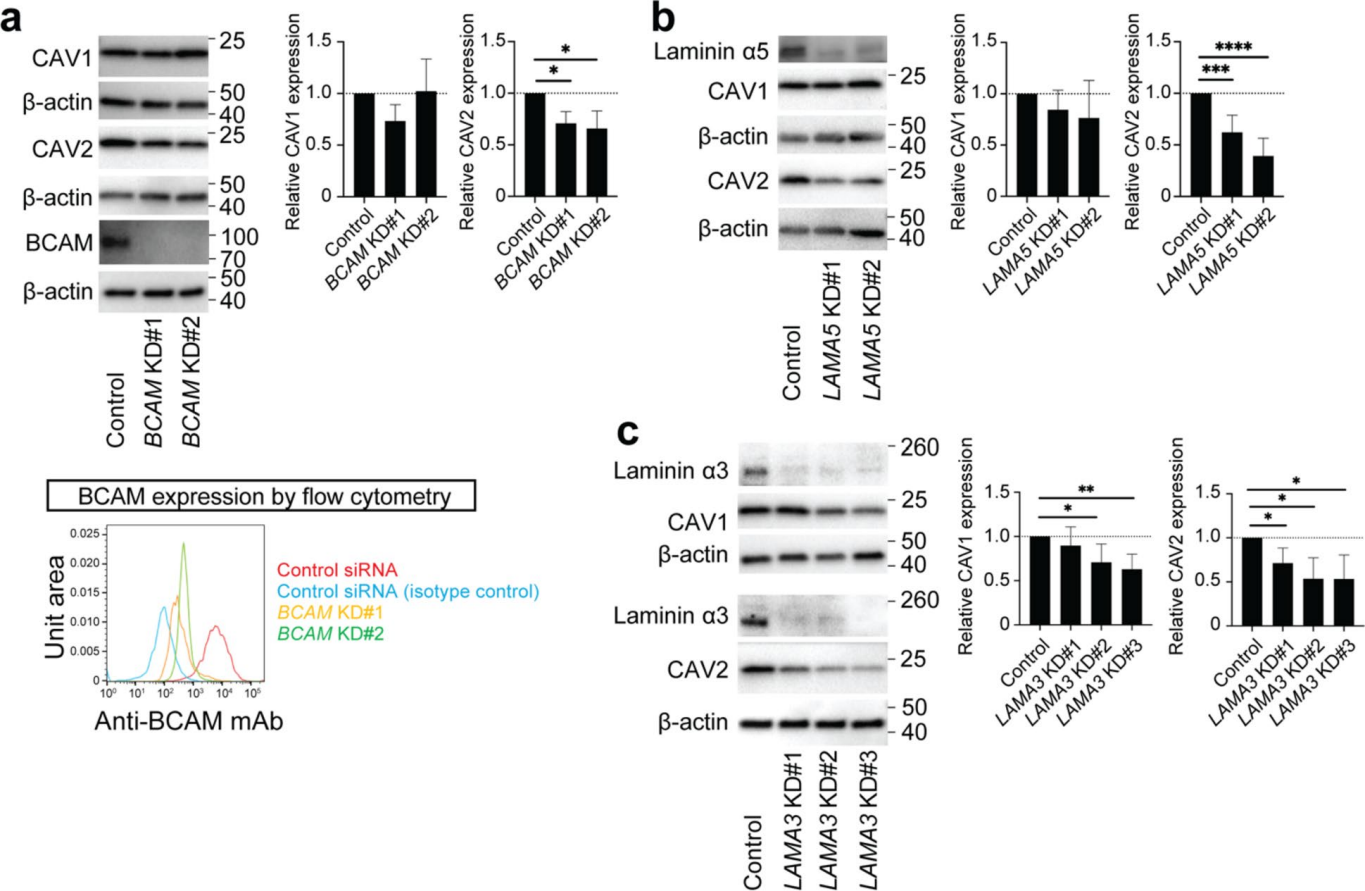
Regulation of CAV1 and CAV2 expression in limbal epithelial cells. (**a**) Left, western blot analysis of CAV1, CAV2 and BCAM expression in *BCAM* KD limbal epithelial cells. Right, bar graph depicts the quantitative analyses of CAV1 and CAV2 protein expression. Bottom, representative flow cytometry analysis of BCAM expression in *BCAM* KD limbal epithelial cells. *n* = 4, *p < 0.05, KD, knockdown. (**b**) Left, western blot analyses of CAV1, CAV2 and laminin α5 expression in *LAMA5* KD limbal epithelial cells. Right, the bar graphs represent the quantitative analyses of CAV1 and CAV2 protein expression. *n* = 8, ***p < 0.001, ****p < 0.0001. (**c**) Left, Western blot analyses of CAV1, CAV2 and laminin α3 expression in control and *LAMA3* KD limbal epithelial cells. Right, the bar graphs represent the quantitative analyses of CAV1 and CAV2 protein expression. *n* = 6, *p < 0.05, **p < 0.01.

Based on the CAV1, CAV2 and BCAM co-expression with Laminin α5 in the limbus and the established role of Laminin α5 as a specific BCAM ligand^28,29^, we hypothesized that CAV1 and CAV2 expression might be dependent on BCAM/Laminin α5 signaling. To test whether BCAM and Laminin α5 contribute to maintaining CAV1 and CAV2 expression in the limbus, we performed siRNA-induced *BCAM* and *LAMA5* knockdown (KD) experiments in human limbal epithelial cells. Both *BCAM* and *LAMA5* KD reduced the expression of CAV2, to 70.9 ± 11.3%, *p* = 0.0232 (*BCAM* KD#1) or 65.8 ± 17.1%, *p* = 0.0457 (*BCAM* KD#2) (*n* = 4), and to 62.2 ± 16.6%, *p* = 0.0007 (*LAMA5* KD#1) or 39.3 ± 17.2%, *p* < 0.0001 (*LAMA5* KD#2) (*n* = 8), respectively, while no significant difference in CAV1 expression was observed (Fig. 2a and b). Based on the Laminin α3, CAV1 and CAV2 co-expression in the basal epithelial layer of the limbus and cornea (Fig. 1d), we also examined whether Laminin α3 played a role in regulating CAV1 and CAV2 expression. SiRNA-induced *LAMA3* KD decreased expression of CAV1 to 89.7 ± 21.1%, *p* = 0.5397 (*LAMA3* KD#1), 70.8 ± 20.8%, *p* = 0.0428 (*LAMA3* KD# 2), or 63.1 ± 17.0%, *p* = 0.0075 (*LAMA3* KD#3) (*n* = 6), and the expression of CAV2, to 71.5 ± 17.0%, *p* = 0.0217 (*LAMA3* KD#1), 53.6 ± 23.8%, *p* = 0.0119 (*LAMA3* KD#2), or 53.4 ± 27.2%, *p* = 0.0201 (*LAMA3* KD#3) (*n* = 6) (Fig. 2c).

### CAV1 and CAV2 Contribute to High Proliferative Capacity of BCAM-positive limbal epithelial cells

**Fig. 3:**
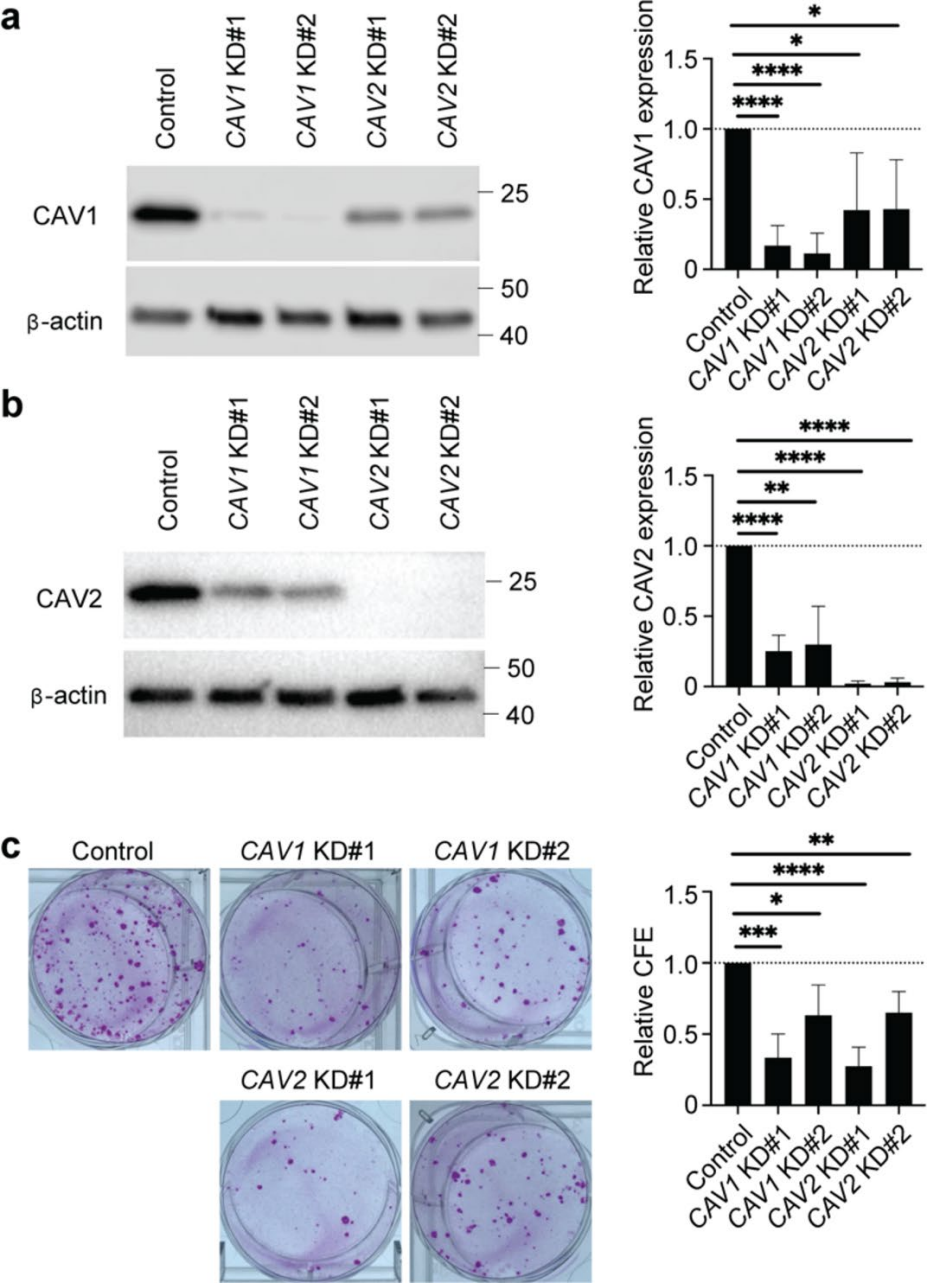
Contribution of CAV1 and CAV2 to colony-forming efficiency. (**a**) Left, western blot analyses of CAV1 expression in *CAV1* and *CAV2* KD limbal epithelial cells. Right, the bar graph depicts quantitative analyses of CAV1 protein expression. *n* = 9. *p < 0.05, ****p < 0.0001, KD, knockdown. (**b**) Left, western blot analyses of CAV2 expression in *CAV1* and *CAV2* KD limbal epithelial cells. Right, the bar graph illustrates quantitative analyses of CAV2 protein expression. *n* = 9. **p < 0.01, ****p < 0.0001. (**c**) Left, representative macroscopic images of the colonies formed by *CAV1* and *CAV2* KD cells compared to the control siRNA transfected cells. Right, the bar graph represents comparative analyses of colony-forming efficiency (CFE). *n* = 7. *p < 0.05, **p < 0.01, ***p < 0.001, ****p < 0.0001.

BCAM-positive limbal epithelial cells are characterized by high proliferative capacity manifested by enhanced colony-forming efficiency (CFE) compared to BCAM-negative cell populations^10^. To test whether CAV1 and CAV2 contribute to enhanced cell proliferation, we examined the CFE of *CAV1* and *CAV2* siRNA KD BCAM-expressing limbal epithelial cell cultures. Western blot analyses confirmed the reduction of CAV1 and CAV2 protein expression in their respective knockdown cells (Fig. 3a and b). Notably, in addition to the loss of CAV1 expression, reduced CAV2 levels were also detected in *CAV1* KD cells (Fig. 3a). Similarly, in *CAV2* KD cells, we observed, in addition to the loss of CAV2, reduced expression of CAV1 (Fig. 3b). Both *CAV1* KD and *CAV2* KD cells demonstrated reduced CFE compared to negative controls, to 33.4 ± 16.8%, *p* = 0.0002 (*CAV1* KD#1) or 63.2 ± 21.4%, *p* = 0.0118 (*CAV1* KD#2), and to 27.6 ± 13.2%, *p* < 0.0001 (*CAV2* KD#1) or 65.2 ± 14.7%, *p* = 0.0024 (*CAV2* KD#2) (*n* = 7) (Fig. 3c). These findings point to a functional role of CAV1 and CAV2 in cell proliferation.

## CAV1 and CAV2 contribute to the maintenance of FGFR2 cell surface expression

**Fig. 4:**
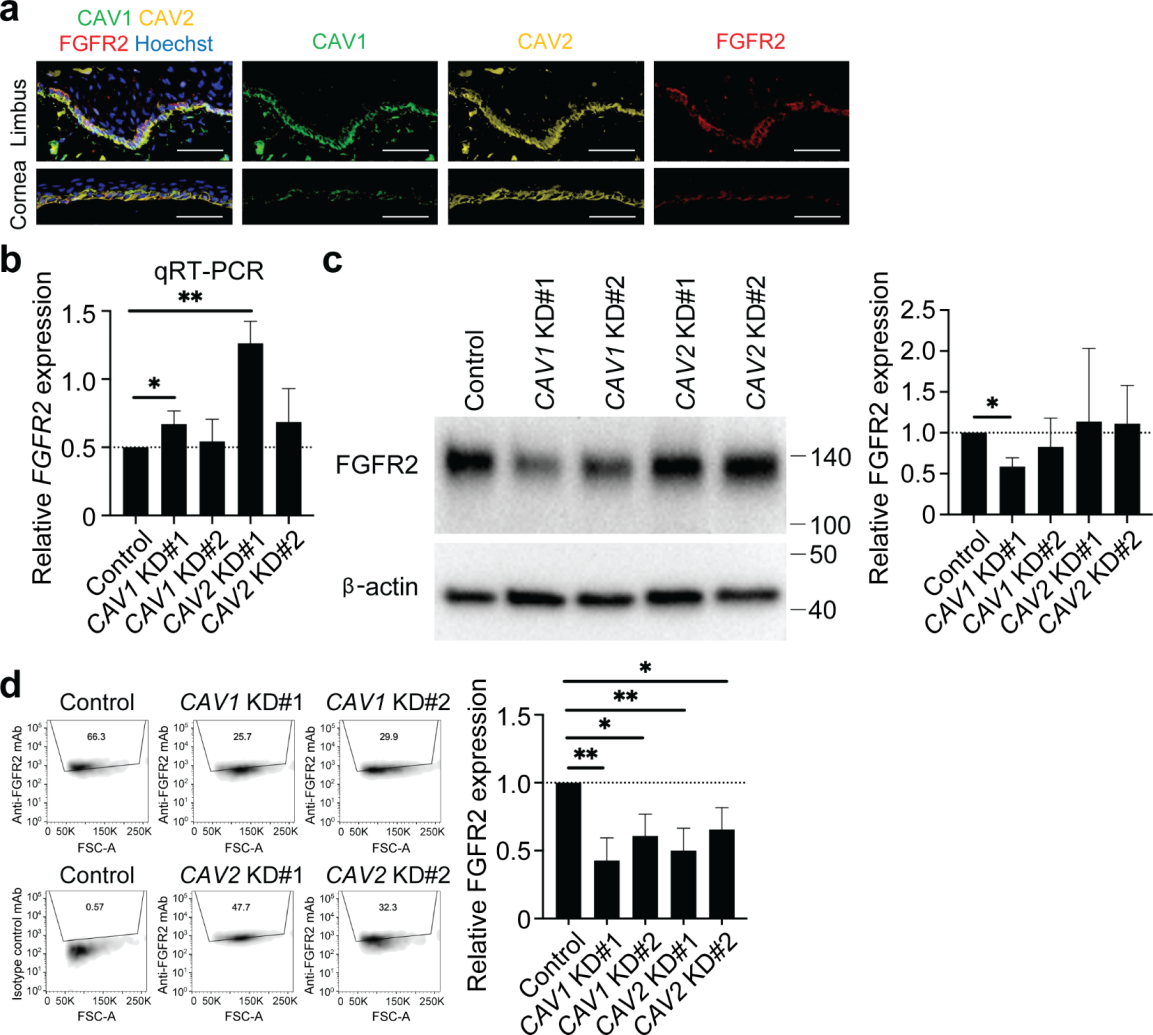
Maintenance of cell surface FGFR2 expression by CAV1 and CAV2. **(a**) Representative immunostaining analyses of CAV1 (green), CAV2 (yellow) and FGFR2 (red) expression in the limbus and cornea. Nuclei are visualized with Hoechst 33342 (blue). *n* = 3. Scale bar, 50 μm. (**b**) Gene expression of *FGFR2* in control and *CAV1* and *CAV2* siRNA-treated limbal epithelial cells. *n* = 5. *p < 0.05, **p < 0.01, KD, knockdown. (**c**) Left, western blot analyses of FGFR2 expression in *CAV1* and *CAV2* KD limbal epithelial cells. Right, bar graph illustrates quantitative analyses of FGFR2 protein expression. *n* = 4. *p < 0.05. (**d**) Left, representative flow cytometry analyses of FGFR2 expression in *CAV1* and *CAV2* KD limbal epithelial cells. Right, the bar graphs represent the quantitative analyses of FGFR2 expression. *n* = 5, *p < 0.05, **p < 0.01, KD, knockdown, FSC, forward scatter; A, area.

Fibroblast growth factor receptors (FGFRs) are abundantly expressed in the caveolae^32,33^. Among them, FGFR2 plays a critical role in the limbus and cornea by contributing to the maintenance of the epithelial phenotype through interaction with its ligand, Fibroblast growth factor 7 (FGF7), also known as keratinocyte growth factor (KGF)^34–36^. Immunohistochemistry of human corneas revealed that FGFR2 was predominantly co-expressed with CAV1 and CAV2 in basal limbal epithelial cells (Fig. 4a). While *CAV1* KD and *CAV2* KD did not affect the *FGFR2* mRNA expression and total FGFR2 protein amount (Fig. 4b and c), flow cytometry analyses showed that *CAV1KD* and *CAV2* KD led to a decrease of cell surface FGFR2 expression when compared to control KD, to 42.8 ± 16.7%, *p* = 0.0044 (*CAV1* KD#1) or 60.8 ± 16.2%, *p* = 0.0155 (*CAV1* KD#2), and to 50.1 ± 16.6%, *p* = 0.0070 (*CAV2* KD#1) or 65.5 ± 16.2%, *p* = 0.0244 (*CAV2* KD#2) (*n* = 5) (Fig. 4d). These results point to a role of CAV1 and CAV2 in the maintenance of FGFR2 expression on the cell surface.

## Discussion

In the current study, we investigated the functional role of CAV1 and CAV2 in the human cornea. We found that CAV1 and CAV2 contribute to the proliferative capacity of BCAM-positive corneal epithelial progenitors at various stages of differentiation^10^. These progenitors form the basal layer of both the limbus and central cornea and are known to be early (KRT12-negative in the limbus) and late (KRT12-positive in the central cornea) TACs. Mechanistically, this could be attributed at least in part to the function of CAV1 and CAV2 in the maintenance of the cell surface expression of the critical regulator of corneal proliferation, FGFR2.

Laminins are a family of extracellular matrix glycoproteins that play a crucial role in the structural scaffolding of basement membranes. Composed of three different chains (α, β and γ), laminins are involved in various biological processes, including cell adhesion, differentiation, migration, and signal transduction^37–40^. In the cornea, Laminin-511 (α5β1γ1) and Laminin-332 (α3β3γ2) are particularly important for the adhesion and migration of corneal epithelial cells and basement membrane integrity^30,31^. Laminins α3 and α5 are distinct subunits of laminin proteins with structural differences in the laminin G-like (LG) domains, which are responsible for the variations in binding affinities for cell surface receptors, such as integrins, dystroglycan, and syndecans^41^. Our immunohistochemical analyses of human corneas revealed that CAV1 and CAV2 were co-expressed with the corneal progenitor marker BCAM^10^ along the basal corneal epithelial layer adjacent to the Laminin α5 and α3-positive basement membrane in the limbus and the Laminin α3-positive basement membrane in the cornea suggesting the potential functional connection. SiRNA-induced *BCAM*, *LAMA5* and *LAMA3* KD resulted in reduced expression of CAV2 and *LAMA3* KD reduced the expression of CAV1. These findings suggest that CAV1 and CAV2 in basal epithelial cells are maintained by specific microenvironmental cues dependent on the intact BCAM, Laminin α5 and α3 signaling axis. Numerous mechanisms of transcriptional regulation of CAV1 expression have been identified to date^42^. Among them, the mitogen-activated protein kinase pathway extracellular signal-regulated kinase (ERK) has been shown to control CAV1 expression^42,43^. Laminin-332 was shown to induce cell proliferation through phosphorylation of the β4 integrin subunit and subsequent activation of ERK^44^. Thus, it is conceivable that Laminin α3 might regulate CAV1 expression through the phosphorylation of ERK. The transcriptional regulation of CAV2 is far less studied. In pancreatic cancer, it has been shown to be controlled by BRD4 and to contribute to cell growth^45^. Another study showed that CAV2 requires co-expression with CAV1 for the formation of a hetero-oligomeric complex between them to be transported from the Golgi to the plasma membrane^24^. This functional dependency might explain our observation of reduced CAV2 expression in the setting of *CAV1* KD.

Our finding of reduced CFE in *CAV1* and *CAV2* KD cultures suggests that CAV1 and CAV2 contribute to cell proliferation. We found that CAV1 and CAV2 maintain FGFR2 expression on the cell surface. FGFR2 is a critical regulator of corneal epithelial proliferation and differentiation, which is triggered by FGFR2 binding to its ligand, FGF7. Furthermore, FGF7 secreted by limbal fibroblasts binds with high affinity to FGFR2 and is reported to be essential for proliferation^34,35,46^ and maintenance of corneal epithelium^36,47–49^. Our results, therefore, suggest that CAV1 and CAV2 contribute to maintaining the proliferative corneal epithelial phenotype in basal epithelial cells through their role in regulating FGFR2 signaling.

In conclusion, our study reveals novel functional roles of CAV1 and CAV2 expressed by BCAM-positive corneal progenitors. Specifically, CAV1 and CAV2 are essential for the corneal progenitor proliferation as a result of their contribution to the maintenance of cell surface FGFR2 expression.

## Materials and methods

### Human cell source

Human whole eye globes and corneas were obtained from consented donors according to Institutional Review Board (IRB)-approved protocols through the Saving Sight (Kansas City, MO) and CorneaGen (Seattle, WA) eye banks. 

All experimental protocols were approved by the Brigham and Women’s Hospital Institutional Review Board committee. The whole eye globes were used for immunostaining. The corneas were used for limbal epithelial cell isolation. Limbal epithelial cells were harvested from the corneas as reported previously^11,50^. Briefly, the central corneas were collected by making circular cuts using an 8 mm disposable biopsy punch (Integra LifeSciences, Plainsboro, NJ), and the corneal endothelium was removed mechanically. Limbal epithelial cells were isolated after 1-hour incubation with PluriSTEM Dispase II Solution (MilliporeSigma, Burlington, MA) at 37 °C and dissociated by TrypLE Express Enzyme (Thermo Fisher Scientific, Waltham, MA) at 37 °C for 30 min. The cells were cultured in DMEM/F12 medium (Thermo Fisher Scientific) supplemented with 10 ng/ml keratinocyte growth factor (KGF) (PeproTech, Rocky Hill, NJ), 10 µM Y-27,632 (Tocris Bioscience, Bristol, UK) and B-27 Supplement (Thermo Fisher Scientific)^47^.

### Rodent Cell source

For colony-forming assay, 3T3-J2 cell line (Kerafast, Boston, MA) was maintained in DMEM (Thermo Fisher Scientific) supplemented with 10% calf serum (GE Healthcare Life Sciences, Marlborough, MA).

## Immunofluorescence staining

Whole globes were fixed with 10% neutral buffered formalin (Fisher Scientific, Pittsburgh, PA) at 4 °C overnight and immersed into 70% ethanol. Paraffin-embedding was performed in BWH Pathology Core. Tissues were cut into 5 μm sections using a microtome. Deparaffinization and antigen retrieval were completed prior to antibody staining procedure. For the staining of FGFR2, the tissues were cryopreserved with the TissueTek^®^ OCT Compound (Sakura, Tokyo, Japan) and 5 μm sections were obtained by cryostat. The fresh frozen sections were fixed with 4% paraformaldehyde (Electron Microscopy Sciences, Hatfield, PA) for 15 min at room temperature before staining. Permeabilization and blocking were performed by a buffer containing 5% normal donkey serum (Jackson ImmunoResearch Laboratories, West Grove, PA) and 0.3% Triton X-100 (MilliporeSigma) for 30 min at room temperature. The sections were subsequently incubated with primary antibodies overnight at 4 °C. The following primary antibodies were used: mouse anti-CAV1 mAb (1:100, Santa Cruz Biotechnology, Santa Cruz, CA) for co-staining with FGFR2, rabbit anti-CAV1 pAb (1:200, GeneTex, Irvine, CA) for co-staining with CAV2, Laminin α5 and Laminin α3, goat anti-CAV2 pAb (1:100, R&D Systems, Minneapolis, MN), rabbit anti-BCAM polyclonal antibody (pAb) (1:100, Novus Biologicals, Centennial, CO), mouse anti-Laminin α5 mAb (1:50, Atlas Antibodies, Bromma, Sweden), mouse anti-Laminin α3 mAb (1:100, Atlas Antibodies), rabbit anti-FGFR2 mAb (1:100, Cell Signaling Technology, Danvers, MA). The sections were washed with Tris-buffered saline (TBS) (Boston BioProducts, Ashland, MA), followed by their incubation with Alexa Fluor 488-conjugated mouse secondary antibody (Abcam, Cambridge, UK), Alexa Fluor 568-conjugated rabbit secondary antibody (Abcam) and Alexa Fluor 647-conjugated goat secondary antibody (Abcam) for 1 h at room temperature and staining with Hoechst 33342 (Thermo Fisher Scientific) for 10 min at room temperature. After being washed with TBS, the sections were sealed with ProLong Gold Antifade Mountant (Thermo Fisher Scientific). Images were taken by C2 + confocal microscope (Nikon, Tokyo, Japan) and analyzed by NIS-Elements AR v4.30.01 (Nikon).

## RNA interference

RNA interference was performed by transfecting *Silencer*™ Select siRNAs (Thermo Fisher Scientific) using Lipofectamine™ RNAiMAX Transfection Reagent (Thermo Fisher Scientific) as previously described^51^. The siRNAs used were: *Silencer*™ Select Negative Control No.1 siRNA, *BCAM* siRNAs (s8336 and s8337), and *LAMA5* siRNAs (s8065 and s8066), *LAMA3* siRNAs (s8059, s8060 and s534951), *CAV1* siRNAs (s2446 and s2448), and *CAV2* siRNAs (s2450 and s2451).

### Western blot analyses

Cultured limbal epithelial cells were dissolved in RIPA buffer (Cell Signaling Technology) supplemented with cOmplete™ Protease Inhibitor Cocktail (MilliporeSigma). The lysates were incubated for 30 min on ice, centrifuged to remove the debris, and its protein concentration was measured by Bio-Rad Protein Assay (Bio-Rad, Hercules, CA). After being mixed with SDS-sample buffer (Boston BioProducts) and 2-mercaptoethanol (MilliporeSigma), the lysates were denatured for 10 min at 95 °C. The proteins were run on SDS-PAGE gel electrophoresis and subsequently transferred on the PVDF blotting membranes (GE Healthcare Life Sciences). The membranes were blocked by a buffer containing 5% blotting-grade blocker (Bio-Rad) for 1 h at room temperature and then incubated with primary antibodies overnight at 4 °C. Primary antibodies used in the current study were: rabbit anti-β-actin pAb (1:1000, Cell Signaling Technology), rabbit anti-CAV1 pAb (1:500, GeneTex), rabbit anti-CAV2 pAb (1:1000, GeneTex), rabbit anti-BCAM pAb (1:1000, ABClonal, Woburn, MA), rabbit anti-laminin α5 pAb (1:500, GeneTex), mouse anti-laminin α3 mAb (1:500, Atlas Antibodies) and rabbit anti-FGFR2 mAb (1:1000, Cell Signaling Technology). After thorough washes with TBS with Tween 20 (MilliporeSigma) (TBS-T), the membranes were incubated with HRP-conjugated mouse or rabbit secondary antibody (Cell Signaling Technology) for 1 h at room temperature. The protein signals were detected using Western Lightning Plus-ECL (PerkinElmer, Waltham, MA), and images were acquired by ChemiDoc MP Imaging System (Bio-Rad). Expression levels of protein were quantified using Image Lab software v5.2.1 (Bio-Rad) and normalized to the expression level of β-actin.

### Flow Cytometry

Dissociated cultured limbal epithelial cells were incubated for 30 min on ice with 4 µg/ml VioBright FITC-conjugated anti-BCAM mAb (Miltenyi Biotec, Bergisch Gladbach, Germany) and 0.57 µg/ml anti-FGFR2 mAb (Cell Signaling Technology). For the detection of FGFR2, donkey anti-rabbit IgG antibody (Alexa Fluor 488) was used as the secondary antibody. GloCell™ Fixable Viability Dye Red 780 (STEMCELL Technologies, Vancouver, Canada) or Propidium Iodide Staining Solution BD Biosciences, San Jose, CA) was applied to remove the dead cells.

Cell analysis was conducted using a FACSCelesta (BD Biosciences) and the data were further analyzed by BD FACSDiva v9.0 and FlowJo v10.5.0 (BD Biosciences).

### Colony-forming assay

Colony-forming assay (CFA) was performed following the protocol previously reported^11,50^. Briefly, trypsinized limbal epithelial cells were seeded on the 3T3-J2 feeder cell layer treated with mitomycin C (MMC) (MilliporeSigma) at 500 cells per well on 6-well plates and were cultured for 10 days in keratinocyte culture medium (KCM) supplemented with 10 ng/ml KGF and 10 µM Y-27632. KCM consists of DMEM without glutamine and Ham’s F-12 Nutrient Mix (Thermo Fisher Scientific) combined in a ratio of 3:1, supplemented with 10% FBS, 0.4µg/ml hydrocortisone hydrogen succinate (MilliporeSigma), 2nM 3,3’,5-triiodo-l-thyronine sodium salt (MilliporeSigma), 1 nM cholera toxin (List Biological Laboratories, Campbell, CA), 2.25 µg/ml bovine transferrin HOLO form (Thermo Fisher Scientific), 2mM L-glutamine (Thermo Fisher Scientific), 0.5% (vol/vol) insulin transferrin selenium solution (Thermo Fisher Scientific), and 1% (vol/vol) penicillin-streptomycin solution GE Healthcare Life Sciences). Formed colonies were fixed with 10% neutral buffered formalin and stained with Rhodamine B (MilliporeSigma). The colony-forming efficiency was calculated by dividing the number of colonies by 500.

### Reverse transcription and quantitative PCR (qPCR)

Extracted total RNA was converted into cDNA by High-Capacity cDNA Reverse Transcription Kit (Thermo Fisher Scientific). qPCR was performed using TaqMan™ Fast Universal PCR Master Mix (Thermo Fisher Scientific) and TaqMan™ Gene Expression Assay probes: *GAPDH* (Hs99999905_m1), *CAV1* (Hs00971716_m1), *CAV2* (Hs00184597_m1) and *FGFR2* (Hs01552918_m1) (Thermo Fisher Scientific). The cycling condition was 95 °C for 20 s and 50 cycles of [95 °C /1 s; 60 °C /20 s] with StepOnePlus™ Real-Time PCR System (Thermo Fisher Scientific). Relative gene expression was calculated with normalization against *GAPDH* as a reference gene.

### Statistical analysis

The data are presented as mean ± standard deviation (SD) and a paired t-test was performed to compare the RNA expression of *CAV1* and *CAV2* between BCAM-positive cells and BCAM-negative cells. Dunnett’s tests were performed to compare the siRNA-treated samples with negative control samples. *p < 0.05, **p < 0.01, ***p < 0.001, ****p < 0.0001.

## Electronic supplementary material

Below is the link to the electronic supplementary material.

Supplementary Material 1

## Data Availability

The datasets used and/or analyzed during the current study available from the corresponding author on reasonable request.

## References

[1] Lehrer, M. S., Sun, T. T. & Lavker, R. M. Strategies of epithelial repair: modulation of stem cell and transit amplifying cell proliferation. *J. Cell. Sci.* **111 (Pt 19)**, 2867–2875. 10.1242/jcs.111.19.2867 (1998). 9730979 10.1242/jcs.111.19.2867

[2] Jongkhajornpong, P. et al. Elevated expression of ABCB5 in ocular surface squamous neoplasia. *Sci. Rep.* **6**, 20541. 10.1038/srep20541 (2016). 26843453 10.1038/srep20541 PMC4740799

[3] Ksander, B. R. et al. ABCB5 is a limbal stem cell gene required for corneal development and repair. *Nature* **511**, 353–357. 10.1038/nature13426 (2014). 25030174 10.1038/nature13426 PMC4246512

[4] Kureshi, A. K., Dziasko, M., Funderburgh, J. L. & Daniels, J. T. Human corneal stromal stem cells support limbal epithelial cells cultured on RAFT tissue equivalents. *Sci. Rep.* **5**, 16186. 10.1038/srep16186 (2015). 26531048 10.1038/srep16186 PMC4632025

[5] Mathan, J. J., Ismail, S., McGhee, J. J., McGhee, C. N. & Sherwin, T. Sphere-forming cells from peripheral cornea demonstrate the ability to repopulate the ocular surface. *Stem Cell Res. Ther.* **7**, 81. 10.1186/s13287-016-0339-7 (2016). 27250558 10.1186/s13287-016-0339-7 PMC4888426

[6] Norrick, A. et al. Process development and safety evaluation of ABCB5(+) limbal stem cells as advanced-therapy medicinal product to treat limbal stem cell deficiency. *Stem Cell Res. Ther.* **12**, 194. 10.1186/s13287-021-02272-2 (2021). 33741066 10.1186/s13287-021-02272-2 PMC7980611

[7] Parfitt, G. J. et al. Immunofluorescence tomography of mouse ocular surface epithelial stem cells and their Niche Microenvironment. *Invest. Ophthalmol. Vis. Sci.* **56**, 7338–7344. 10.1167/iovs.15-18038 (2015). 26559480 10.1167/iovs.15-18038 PMC4642609

[8] Shaharuddin, B., Ahmad, S., Md Latar, N., Ali, S. & Meeson, A. A. Human corneal epithelial cell line model for Limbal Stem Cell Biology and Limbal Immunobiology. *Stem Cells Transl. Med.* **6**, 761–766. 10.5966/sctm.2016-0175 (2017). 28297580 10.5966/sctm.2016-0175 PMC5442771

[9] Shaharuddin, B. et al. Human limbal mesenchymal stem cells express ABCB5 and can grow on amniotic membrane. *Regen. Med.* **11**, 273–286. 10.2217/rme-2016-0009 (2016). 26965478 10.2217/rme-2016-0009

[10] Sasamoto, Y. et al. Limbal BCAM expression identifies a proliferative progenitor population capable of holoclone formation and corneal differentiation. *Cell Rep.* **40**, 111166. 10.1016/j.celrep.2022.111166 (2022). 35947947 10.1016/j.celrep.2022.111166 PMC9480518

[11] Sasamoto, Y., Yeung, P. C., Tran, J., Frank, M. H. & Frank, N. Y. Protocol for isolating human BCAM-positive corneal progenitor cells by flow cytometry and cell sorting. *STAR Protoc.* **4**, 102503. 10.1016/j.xpro.2023.102503 (2023). 37669162 10.1016/j.xpro.2023.102503 PMC10485628

[12] Cohen, A. W., Hnasko, R., Schubert, W. & Lisanti, M. P. Role of caveolae and caveolins in health and disease. *Physiol. Rev.* **84**, 1341–1379. 10.1152/physrev.00046.2003 (2004). 15383654 10.1152/physrev.00046.2003

[13] Rothberg, K. G. et al. Caveolin, a protein component of caveolae membrane coats. *Cell* **68**, 673–682. 10.1016/0092-8674(92)90143-z (1992). 1739974 10.1016/0092-8674(92)90143-z

[14] Pelkmans, L. & Helenius, A. Endocytosis via caveolae. *Traffic* **3**, 311–320. 10.1034/j.1600-0854.2002.30501.x (2002). 11967125 10.1034/j.1600-0854.2002.30501.x

[15] Shaul, P. W. & Anderson, R. G. Role of plasmalemmal caveolae in signal transduction. *Am. J. Physiol.* **275**, L843–851. 10.1152/ajplung.1998.275.5.L843 (1998). 9815100 10.1152/ajplung.1998.275.5.L843

[16] Fielding, C. J. & Fielding, P. E. Caveolae and intracellular trafficking of cholesterol. *Adv. Drug Deliv. Rev.* **49**, 251–264. 10.1016/s0169-409x(01)00140-5 (2001). 11551398 10.1016/s0169-409x(01)00140-5

[17] Amino, K., Honda, Y., Ide, C. & Fujimoto, T. Distribution of plasmalemmal ca(2+)-pump and caveolin in the corneal epithelium during the wound healing process. *Curr. Eye Res.* **16**, 1088–1095. 10.1076/ceyr.16.11.1088.5098 (1997). 9395767 10.1076/ceyr.16.11.1088.5098

[18] Couet, J., Sargiacomo, M. & Lisanti, M. P. Interaction of a receptor tyrosine kinase, EGF-R, with caveolins. Caveolin binding negatively regulates tyrosine and serine/threonine kinase activities. *J. Biol. Chem.* **272**, 30429–30438. 10.1074/jbc.272.48.30429 (1997). 9374534 10.1074/jbc.272.48.30429

[19] García-Cardeña, G. et al. Dissecting the interaction between nitric oxide synthase (NOS) and caveolin. Functional significance of the nos caveolin binding domain in vivo. *J. Biol. Chem.* **272**, 25437–25440. 10.1074/jbc.272.41.25437 (1997). 9325253 10.1074/jbc.272.41.25437

[20] Gu, X., Reagan, A. M., McClellan, M. E. & Elliott, M. H. Caveolins and caveolae in ocular physiology and pathophysiology. *Prog. Retin. Eye Res.* **56**, 84–106. 10.1016/j.preteyeres.2016.09.005 (2017). 27664379 10.1016/j.preteyeres.2016.09.005 PMC5237608

[21] Li, S., Couet, J. & Lisanti, M. P. Src tyrosine kinases, Galpha subunits, and H-Ras share a common membrane-anchored scaffolding protein, caveolin. Caveolin binding negatively regulates the auto-activation of src tyrosine kinases. *J. Biol. Chem.* **271**, 29182–29190. 10.1074/jbc.271.46.29182 (1996). 8910575 10.1074/jbc.271.46.29182 PMC6687395

[22] Li, S. et al. Evidence for a regulated interaction between heterotrimeric G proteins and caveolin. *J. Biol. Chem.* **270**, 15693–15701. 10.1074/jbc.270.26.15693 (1995). 7797570 10.1074/jbc.270.26.15693

[23] Scherer, P. E. et al. Cell-type and tissue-specific expression of caveolin-2. Caveolins 1 and 2 co-localize and form a stable hetero-oligomeric complex in vivo. *J. Biol. Chem.* **272**, 29337–29346. 10.1074/jbc.272.46.29337 (1997). 9361015 10.1074/jbc.272.46.29337

[24] Parolini, I. et al. Expression of caveolin-1 is required for the transport of caveolin-2 to the plasma membrane. Retention of caveolin-2 at the level of the golgi complex. *J. Biol. Chem.* **274**, 25718–25725. 10.1074/jbc.274.36.25718 (1999). 10464309 10.1074/jbc.274.36.25718

[25] Mora, R. et al. Caveolin-2 localizes to the golgi complex but redistributes to plasma membrane, caveolae, and rafts when co-expressed with caveolin-1. *J. Biol. Chem.* **274**, 25708–25717. 10.1074/jbc.274.36.25708 (1999). 10464308 10.1074/jbc.274.36.25708

[26] Fujimoto, T., Kogo, H., Nomura, R. & Une, T. Isoforms of caveolin-1 and caveolar structure. *J. Cell Sci.* **113 Pt 19**, 3509–3517 (2000). 10984441 10.1242/jcs.113.19.3509

[27] Rhim, J. H., Kim, J. H., Yeo, E. J., Kim, J. C. & Park, S. C. Caveolin-1 as a novel indicator of wound-healing capacity in aged human corneal epithelium. *Mol. Med.* **16**, 527–534. 10.2119/molmed.2010.00046 (2010). 20644900 10.2119/molmed.2010.00046 PMC2972400

[28] Kikkawa, Y., Moulson, C. L., Virtanen, I. & Miner, J. H. Identification of the binding site for the Lutheran blood group glycoprotein on laminin alpha 5 through expression of chimeric laminin chains in vivo. *J. Biol. Chem.* **277**, 44864–44869. 10.1074/jbc.M208731200 (2002). 12244066 10.1074/jbc.M208731200

[29] Parsons, S. F. et al. Lutheran blood group glycoprotein and its newly characterized mouse homologue specifically bind alpha5 chain-containing human laminin with high affinity. *Blood* **97**, 312–320. 10.1182/blood.v97.1.312 (2001). 11133776 10.1182/blood.v97.1.312

[30] Polisetti, N. et al. Laminin-511 and – 521-based matrices for efficient ex vivo-expansion of human limbal epithelial progenitor cells. *Sci. Rep.* **7**, 5152. 10.1038/s41598-017-04916-x (2017). 28698551 10.1038/s41598-017-04916-x PMC5506065

[31] Ljubimov, A. V. et al. Human corneal basement membrane heterogeneity: topographical differences in the expression of type IV collagen and laminin isoforms. *Lab. Invest.* **72**, 461–473 (1995). 7723285

[32] Bryant, M. R., Marta, C. B., Kim, F. S. & Bansal, R. Phosphorylation and lipid raft association of fibroblast growth factor receptor-2 in oligodendrocytes. *Glia* **57**, 935–946. 10.1002/glia.20818 (2009). 19053057 10.1002/glia.20818 PMC2682628

[33] Feng, L. et al. Caveolin-1 orchestrates fibroblast growth factor 2 signaling control of angiogenesis in placental artery endothelial cell caveolae. *J. Cell. Physiol.* **227**, 2480–2491. 10.1002/jcp.22984 (2012). 21830216 10.1002/jcp.22984 PMC3248968

[34] Wilson, S. E., Walker, J. W., Chwang, E. L. & He, Y. G. Hepatocyte growth factor, keratinocyte growth factor, their receptors, fibroblast growth factor receptor-2, and the cells of the cornea. *Invest. Ophthalmol. Vis. Sci.* **34**, 2544–2561 (1993). 8392040

[35] Sotozono, C., Inatomi, T., Nakamura, M. & Kinoshita, S. Keratinocyte growth factor accelerates corneal epithelial wound healing in vivo. *Invest. Ophthalmol. Vis. Sci.* **36**, 1524–1529 (1995). 7601632

[36] Cheng, C. C., Wang, D. Y., Kao, M. H. & Chen, J. K. The growth-promoting effect of KGF on limbal epithelial cells is mediated by upregulation of DeltaNp63alpha through the p38 pathway. *J. Cell Sci.* **122**, 4473–4480. 10.1242/jcs.054791 (2009). 19920075 10.1242/jcs.054791

[37] Miner, J. H. & Yurchenco, P. D. Laminin functions in tissue morphogenesis. *Annu. Rev. Cell Dev. Biol.* **20**, 255–284. 10.1146/annurev.cellbio.20.010403.094555 (2004). 15473841 10.1146/annurev.cellbio.20.010403.094555

[38] Spenle, C., Simon-Assmann, P., Orend, G. & Miner, J. H. Laminin alpha5 guides tissue patterning and organogenesis. *Cell Adh Migr.* **7**, 90–100. 10.4161/cam.22236 (2013). 23076210 10.4161/cam.22236 PMC3544791

[39] Yap, L., Tay, H. G., Nguyen, M. T. X., Tjin, M. S. & Tryggvason, K. Laminins in Cellular differentiation. *Trends Cell Biol.* **29**, 987–1000. 10.1016/j.tcb.2019.10.001 (2019). 31703844 10.1016/j.tcb.2019.10.001

[40] Givant-Horwitz, V., Davidson, B. & Reich, R. Laminin-induced signaling in tumor cells. *Cancer Lett.* **223**, 1–10. 10.1016/j.canlet.2004.08.030 (2005). 15890231 10.1016/j.canlet.2004.08.030

[41] Matsunuma, M. et al. Chain-specificity of laminin alpha1-5 LG45 modules in the recognition of carbohydrate-linked receptors and intramolecular binding. *Sci. Rep.* **13**, 10430. 10.1038/s41598-023-37533-y (2023). 37369727 10.1038/s41598-023-37533-y PMC10300086

[42] Forbes, A. et al. The tetraspan protein EMP2 regulates expression of caveolin-1. *J. Biol. Chem.* **282**, 26542–26551. 10.1074/jbc.M702117200 (2007). 17609206 10.1074/jbc.M702117200

[43] Engelman, J. A. et al. Recombinant expression of caveolin-1 in oncogenically transformed cells abrogates anchorage-independent growth. *J. Biol. Chem.* **272**, 16374–16381. 10.1074/jbc.272.26.16374 (1997). 9195944 10.1074/jbc.272.26.16374

[44] Kato, K. et al. Plasma-membrane-associated sialidase (NEU3) differentially regulates integrin-mediated cell proliferation through laminin- and fibronectin-derived signalling. *Biochem. J.* **394**, 647–656. 10.1042/BJ20050737 (2006). 16241905 10.1042/BJ20050737 PMC1383714

[45] Jiao, F. et al. Caveolin-2 is regulated by BRD4 and contributes to cell growth in pancreatic cancer. *Cancer Cell Int.* **20**, 55. 10.1186/s12935-020-1135-0 (2020). 32099528 10.1186/s12935-020-1135-0 PMC7029443

[46] Li, D. Q. & Tseng, S. C. Differential regulation of keratinocyte growth factor and hepatocyte growth factor/scatter factor by different cytokines in human corneal and limbal fibroblasts. *J. Cell. Physiol.* **172**, 361–372. https://doi.org/10.1002/(sici)1097-4652(199709)172:3<361::Aid-jcp10>3.0.Co;2-9 (1997). 10.1002/(SICI)1097-4652(199709)172:3<361::AID-JCP10>3.0.CO;2-9 9284956

[47] Miyashita, H. et al. Long-term maintenance of limbal epithelial progenitor cells using rho kinase inhibitor and keratinocyte growth factor. *Stem Cells Transl. Med.* **2**, 758–765. 10.5966/sctm.2012-0156 (2013). 23981725 10.5966/sctm.2012-0156 PMC3785260

[48] Hayashi, M., Hayashi, Y., Liu, C. Y., Tichelaar, J. W. & Kao, W. W. Over expression of FGF7 enhances cell proliferation but fails to cause pathology in corneal epithelium of Kerapr-rtTA/FGF7 bitransgenic mice. *Mol. Vis.* **11**, 201–207 (2005). 15788998

[49] Wilson, S. E. et al. Effect of epidermal growth factor, hepatocyte growth factor, and keratinocyte growth factor, on proliferation, motility and differentiation of human corneal epithelial cells. *Exp. Eye Res.* **59**, 665–678. 10.1006/exer.1994.1152 (1994). 7698260 10.1006/exer.1994.1152

[50] Sasamoto, Y. et al. Investigation of factors associated with ABCB5-positive limbal stem cell isolation yields from human donors. *Ocul. Surf.* **18**, 114–120. 10.1016/j.jtos.2019.10.009 (2020). 31655212 10.1016/j.jtos.2019.10.009 PMC7840156

[51] Fujimoto, S. et al. KLF4 prevents epithelial to mesenchymal transition in human corneal epithelial cells via endogenous TGF-β2 suppression. *Regen. Ther.* **11**, 249–257. 10.1016/j.reth.2019.08.003 (2019). 31538102 10.1016/j.reth.2019.08.003 PMC6745437

