# Supplementary figures and images for: Caveolin 1 and 2 enhance the proliferative capacity of BCAM-positive corneal progenitors
Source: Sci Rep. 2025 Feb 24;15:6672. doi: 10.1038/s41598-024-81283-4 (PMC11850879; doi:10.1038/s41598-024-81283-4)

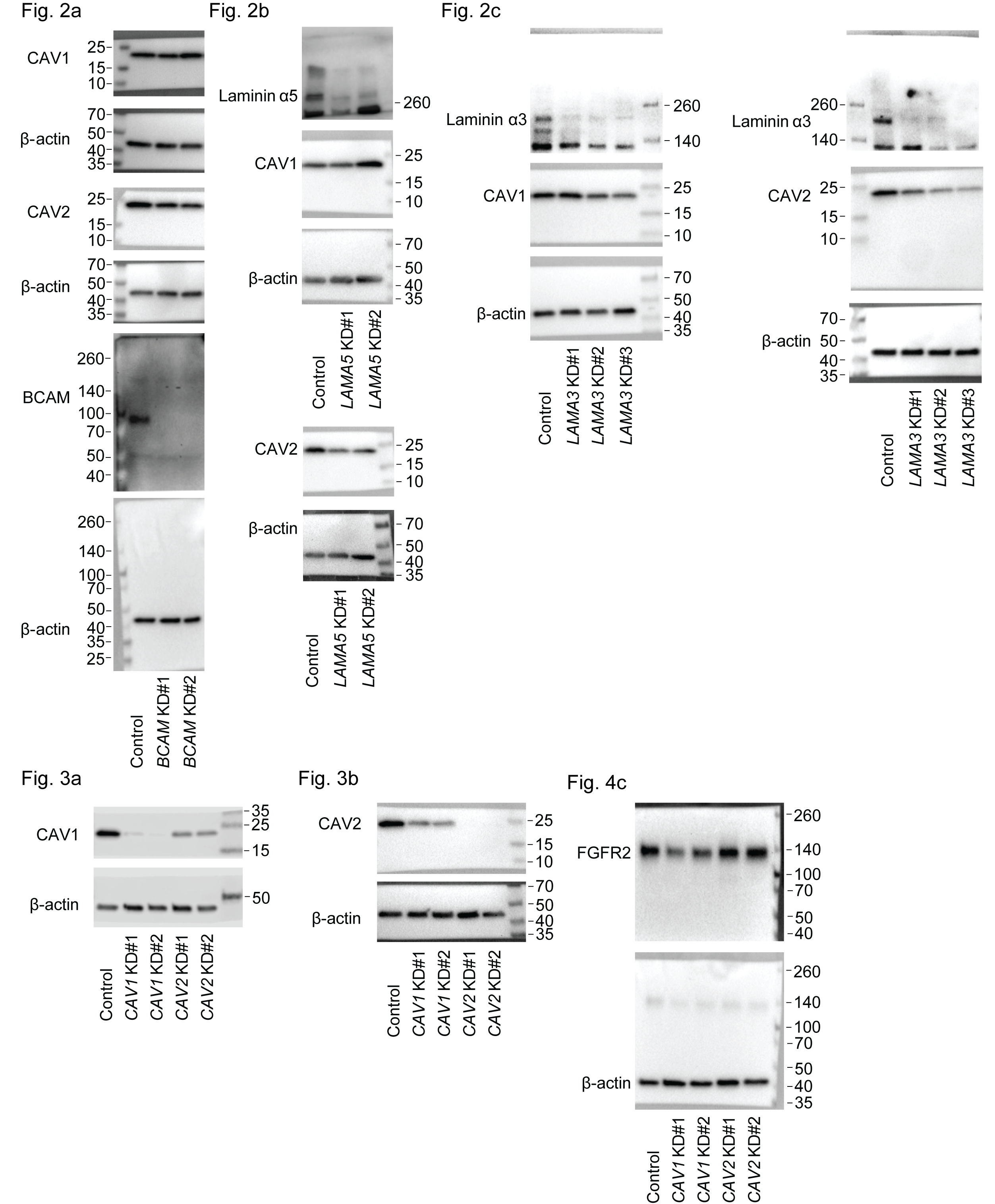

Supplement: Supplementary file 1 — Supplementary Material 1 [file 41598_2024_81283_MOESM1_ESM.tiff]
